# Notes and News

**Published:** 1887-03-19

**Authors:** 


### NOTES AND NEWS.

COLLECTED AND COMMUNICATED.

THE Solicitor-General, Sir Edward Clarke, M.P., Q.C., has promised to preside at the thirty-first annual festival of the Royal Hospital for Incurables, Putney, to be held at the Albion Tavern, Aldersgate Street, on 10th May.

THE wife of the Indian Viceroy is promoting a Jubilee Fund in aid of the National Association for Supplying Female Medical Aid to the Women in India. The Mercers' Company have headed the list with the munificent donation of five hundred guineas.

MISS RAINSFORD, the Lady Superintendent at Lowestoft Hospital, has just been selected out of the many applicants for the appointment of Matron to the Great Northern Central Hospital, London.

THE meeting of the Hospitals Association on Wednesday evening last was well attended, and Dr. Bristowe, F.R.S., who presided, as well as Mr. Samuel Benton, the reader of the paper (which will be found in another column), must have been well satisfied with the proceedings. We hope they will result in much greater public interest in the work of that excellent institution, the Workhouse Nursing Association, of which Miss Wilson is the energetic secretary.

THE funds of the Victoria Hospital for Children are to be benefited by the proceeds of a concert to be held at the Chelsea Town Hall on the 29th inst. Some very distinguished names are programmed, including that of Mr. Sims Reeves, so that the concert will doubtless be largely attended.

A LITTLE timely succour has at length arrived in aid of the Great Northern Central Hospital. The rebuilding of the institution is a vast undertaking, which will probably need several years to accomplish. The Baroness Burdett-Coutts, who is a warm supporter of this charity, held the other afternoon a preliminary meeting at No. 1, Stratton Street, Piccadilly, for the purpose of initiating a fund for erecting free of debt a " Victoria Jubilee Wing," to be exclusively reserved for the reception of women and children. A small central committee, of which the Baroness consented to accept the presidency, was formed, and sub-committees are being created in the northern districts with a view to carrying out the desired object. The hon. secs. are Mrs. A. D. Harkness, St. Mary's Road, N., and Miss Amy Hill, 5, Hyde Park Mansions, W.

ON Friday last the inhabitants of Chester presented Dr. Waters with a fine oil portrait, executed by Frank Holl, and a cheque for £296, in recognition of his eminent services. Dr. Waters is one of the only four recipients of the gold medal of the British Medical Association, awarded to him for his long and self-denying services in the cause of medical reform. The presentation was made by the Duke of Westminster.

WE cull the following from *The World*:—" A very remarkable operation has just been performed by one of the surgeons at the West London Hospital. A child was brought in having a large mole covering nearly the whole of its cheek. He 'transplanted' the mole 'by exchange'; that is, he moved the mole from the cheek to the arm, and planted flesh from the arm to the cheek. Everything succeeded perfectly. Mole and child are doing well."

THE East London Nursing Society is not, as Sir Edmund Currie remarked at the recent annual meeting, a mushroom institution. Established in 1868, for the purpose of providing trained nurses for the sick poor at their own home, the Society has now expanded itself to the very tether of its resources. The denizens of Wapping and its *entourage* want help;—they can give but little; so it comes about that the East London Nursing Society has to depend for its existence upon charitable friends outside the sphere of its own operations. Except its more immediate friends, there are few among the richer classes who know the good this Society does, and the devotion of its little staff of nurses and matrons. We are, therefore, deeply sorry to learn that the Society is so hard pressed for funds; we fully believe that, if its work in the poor East could be laid bare to the wealthy West, such a state of things would not much longer exist. Mr. A. Lacey, of 49, Philpot Street, Commercial Road, E., is the Secretary of the Society.

OUR hospitals reveal some curious incidents at times. Some years ago, at the Cancer Hospital at Brompton, a patient, supposed to be wholly without means, died. In his clothing the officials found, after his death, something like £300. The friends of the deceased quickly put in their claim. From the Consumption Hospital at Brompton comes another strange story. Many years ago a poor man sought and obtained at a farmhouse at Great Horkesley, in Essex, a situation as labourer. The tenants of the farm had a tiny granddaughter, of whom the poor man became very fond. After working for some years in the village, the workman came to London. The little girl reached woman's estate, only to be the victim of that dire disease—consumption. She was accordingly sent to the hospital at Brompton, and there she has just died, but not before she had once more seen her old friend and obtained from him a promise that he would bury her in her native village. The poor workman himself made her coffin. The body being placed in it, was covered in oilskin, put on a truck, and pushed by the girl's old friend along fifty miles of the road to the little village of Great Horkesley, where it has been interred.

The following vacancies are announced this week, the amount of the salary being placed in each case after the name of the institution :—

**Resident Medical Officers.**

Birmingham General Hospital. Surgeon, doubly-qualified.—£130.
Cheltenham General Hospital. House-Surgeon, doubly-qualified.—£80.
Derbyshire Infirmary. Assistant House-Surgeon, qualified.—£10 bonus.
Fisherton Asylum. Assistant, doubly-qualified.—£100.
Royal Free Hospital, W.C. Junior, doubly-qualified.
Royal Portsmouth Hospital. Assistant House-Surgeon, qualified.—£60.
Southampton Royal Infirmary. Assistant. Fourth year's student, at least.
York County Hospital. Assistant House-Surgeon, doubly-qualified.—£50.

**Non-resident Medical Officers.**

Bedford Central Dispensary. One, doubly-qualified.
Easingwold Union. District Officer, doubly-qualified.—£26 and fees.
Manchester Children's Dispensary. One, doubly-qualified.—£180.
Wandsworth Board of Works. Officer of Health for Putney, doubly-qualified.—£75.

**Superintendent.**

Royal Free Hospital, W.C. Lady Superintendent.

**Trained Nurses.**

Bolton Infirmary. Charge, night.—£25.
Eastbourne Nursing Institution. One Monthly.
North-West London Hospital. Night.—£24.
Thrapston Union. One.—£20.
Workington Infirmary. Night.—£18.

**Probationers.**

Bolton Infirmary. One.
Eastbourne Nursing Institution. One.
Saffron Walden Hospital. Lady.

---

Mr. H. Elwin Harris, B.A. Cantab., M.R.C.S., L.R.C.P., has been appointed Assistant Medical Superintendent and Dispenser at the Paddington Infirmary, *vice* Mr. T. Ernest Hillier, M.B., resigned.

---

As an authority upon hospitals, Sir Andrew Clark takes a position of the very highest eminence. We may therefore rest assured that an institution which he commends is one which deserves well of the benevolent. The East London Hospital for Children and Dispensary for Women has been described by Sir Andrew as a model hospital, a title which, in our opinion, it well deserves. Situated away in the far east of London, in the poor and crowded district of Shadwell, the hospital is probably known to but very few of the rich inhabitants of the West. The hospital was founded, under somewhat romantic circumstances, during the cholera epidemic of 1868, and in 1877 the present picturesque edifice was reared. During its career of nineteen years it has afforded relief to 162,632 patients. Its wards are always full, while many of the applicants who crowd the small out-patient department have to be turned away for want of room. To say that children are happier in the hospitals than in their homes might perhaps raise a smile of incredulity, but at the East London this is often actually the fact. The Bishop of Bedford remarked, at a special meeting held at the Mansion House last Friday, in aid of the hospital, that during a recent visit which he paid, he learnt so comfortable are the little children made, that when their recovery has been accomplished, they are loth to leave! When one looks at the wretched waifs and strays—shoeless, dirt-begrimed, and ragged—who swarm in the streets of the Tower Hamlets, and then turns in at the East London Hospital and sees the order, the cleanliness, the clean and happy faces of the little ones, and listens to the kind words of "nursie," he need not wonder that many a time the child would, if it could, stay at the hospital instead of returning to a squalid tenement, which it would be a perversion of words to call "a home."

---

The Royal London Ophthalmic Hospital, in Blomfield Street, Moorfields, which was founded in 1804, ranks as the oldest hospital of its kind in England, and may be regarded as the parent of all the eye infirmaries throughout the kingdom. The admission to this institution is entirely free to the necessitous poor, regardless of nationality or denomination. Last year 28,260 persons received the benefits of this charity, the in-patients numbering 2,049, and the out-patients 26,211. In 1,444 cases spectacles were supplied free of cost, while 190 patients received artificial eyes. At the recent annual meeting of the supporters of this hospital a vote of condolence to Mrs. Streatfield was unanimously passed. The late Mr. J. F. Streatfield, F.R.C.S., who for thirty years was an esteemed and able member of the surgical staff, was a gentleman of very high attainments in his profession, and a warm friend of the poor.

---

Inasmuch as the whole end and object of this Journal, nay, the very reason of its existence, lies in the hope of impressing upon the community the strong claims of our medical charities for support, it is most satisfactory to be able to give honest and unqualified praise to any particular institution. In so doing with respect to the Bristol Hospital for Sick Children and Women, whose report for 1886 has lately reached us, we must not however be understood to imply censure upon other institutions. We speak in this instance from actual personal knowledge, and it is with sincere pleasure that we note the healthy financial condition of the Bristol Hospital for Sick Children. The income for the year 1886 covered the expenditure and left a small balance of £18 to be brought forward for the expenses of the current twelve months. The debt incurred by the recent erection of new buildings has been reduced during 1886 by two gifts of £1,000 and £500 respectively, and now amounts to a trifle under £2,000. This balance is the only cause of anxiety to the committee, and an earnest hope is expressed that generous friends in Bristol and elsewhere will assist in clearing it off during the present year. The total number of beds is ninety-two, one ward with five cots being specially devoted to the treatment of children suffering from ophthalmic affections. All the beds in the ordinary wards have been generally full, 584 children and 29 women having been received as in-patients, a fact which amply justifies the steps taken by the committee for adding to the former accommodation, and so increasing the usefulness of the hospital. The working-classes in Bristol have testified, by subscriptions both from individuals and benefit societies, to their appreciation of the help rendered to their children, a satisfactory sign of the times which will be especially welcome to all who are interested in hospitals and their great work.

THE following sums have been recently received by the various Medical Charities, the name of the donor or the source from which they were obtained being given in each instance after the name of the Institution :—

**Donations.**

Guy's Hospital. The Cordwainers' Company, £105 (towards the Special Fund).
Chelsea Hospital for Women, Fulham Road. W. A., £10. D. G., £10 10s.
North London Hospital. Mr. Rickman J. Godlee, £10. Right Hon. Lord Cranborne, £10.
Home for Incurable Children, Maida Vale. Miss E. Harrold, £10 10s.
Charing Cross Hospital. Rev. F. Jacox, £31 10s. Miss Wedgwood, £10. Mr. W. H. Yatman, £50.
North-Eastern Hospital for Children, Hackney Road. Mutual Friendly Aid Society, per Mr. W. T. Herring, £10 10s.
Dental Hospital of London, Leicester Square. Mr. F. J. Barnett, £10 10s. Mr. Henry A. Brassey, £50 (Jubilee offering).
British Lying-in Hospital. Messrs. Hoare, £21. Miss Richards, £10 10s.
Royal Free Hospital. The Governors of the London Assurance Corporation, £10 10s.
London Temperance Hospital. Two Friends, per Mr. John Taylor, £500.
Royal Ear Hospital. Mercers' Company, £10 10s.
British Home for Incurables. Governors of the London Assurance Corporation, £10 10s.
Royal Hospital for Incurables, Putney. Collected by the Misses Joslin, £21.

**Legacies.**

North London Hospital. Mrs. C. E. T. Holmes, £1,000.
Great Northern Central Hospital. Mrs. C. E. T. Holmes, £1,000.
London Hospital. Mrs. C. E. T. Holmes, £1,000. Mr. Geo. Hawkins, £500.
King's College Hospital. Mrs. C. E. T. Holmes, £1,000.
Mrs. Gladstone's Convalescent Home for the Poor. Mrs. C. E. T. Holmes, £500.
Hospital for Consumption, Brompton. Mrs. C. E. T. Holmes, £500.
Royal Hospital for Incurables, Putney. Mrs. C. E. T. Holmes, £500. Mr. Geo. Hawkins, £100.
West London Hospital. Mrs. C. E. T. Holmes, £500.
Victoria Park Hospital for Diseases of the Chest. Mr. Geo. Hawkins, £100.
London Dispensary, Spitalfields. Mr. Geo. Hawkins, £50.
City of London Truss Society. Mr. Geo. Hawkins, £50.
Royal London Ophthalmic Hospital. Mr. Geo. Hawkins, £50.
Home for Confirmed Invalids, Highbury. Miss Lucy Forster, £300.

---

CHILDREN always evoke our sympathy. In health, their helplessness, their dependence upon, and their trust in us, appeal to the nobler qualities of our nature; in sickness the appeal is intensified a thousandfold, and we hasten to extend the hand of mercy to them. It seems, therefore, somewhat strange that, until comparatively recent years, this country should have possessed no institutions specially devoted to the study and treatment of diseases incidental to childhood. Down to the middle of the present century there were no children's hospitals, and in the general hospitals the little ones were mostly treated in the same wards as the adults. At the West London Hospital, for instance, this was the case till very recently. The reign of the Queen has been fruitful of grand institutions, foremost among which we must certainly place the establishment of children's hospitals. Great accomplishments frequently have very small commencements. This was especially the case in connection with the foundation of the first children's hospital—that in Great Ormond Street. The Hospital for Sick Children was established by Dr. West in the year 1852, in an old-fashioned mansion, the residence, a century before, of that famous physician and "archiator," Dr. Richard Mead. The hospital started with a single cot, whose first tenant was a little girl. Gradually the accommodation was increased. At the end of the first month we find that eight in-patients and twenty-four out-patients had been treated by the benevolent founder. In the first annual report we are told that the Queen had consented to become the patroness of the hospital. Charles Dickens, an ardent lover of little children, was a staunch friend of the Great Ormond Street Hospital, and the first dinner in aid of the hospital (at which he presided) resulted in the sum of £3,000 being raised in a single evening. The subsequent history of this admirable institution is too well known to need recapitulation here. The present enlarged hospital is, as everyone knows, in a very unfinished state. As the institution is the oldest children's hospital, and is essentially identified with Queen Victoria's reign, it has occurred to the Council that the present was an auspicious occasion for completing and endowing the hospital by means of a Children's Jubilee Tribute. A sum of £16,000 is required to complete the south wing and fit it for the reception of little patients, an amount which might easily be raised if the little children of our well-to-do classes throughout England, Scotland, and Ireland were each to send a few pence. There is one fact which it is important to bear in mind. The hospital in Great Ormond Street is not only the means of annually restoring to health some 16,000 little ones, but a vehicle for doing good on a much more extended scale. The institution presents a very wide field for the study of almost every form of infantile disease, and the larger insight, the increased knowledge acquired by the opportunities of study there afforded to our doctors, are facts of which we ought not to lose sight.

---

INSTITUTIONS for nursing the sick poor at their own homes, provided they are efficiently conducted, constitute most useful adjuncts to our hospitals. Skilled nursing is quite as essential as medical treatment; indeed, the latter without the former is practically valueless. The North London Nursing Association for the Sick Poor, which has been in existence now for six years, is doing, in a poor and thickly populated district, a work of the highest utility; and that, too, in spite of a very impoverished exchequer. The Society employs eight nurses, a number which is quite inadequate for the amount of work which is cast upon them. It is not therefore surprising to learn that, during the past year, one or another of this little band had herself to lie by on a bed of sickness. During 1886, the cases nursed numbered 1,102, involving no less than 22,224 visits, performed at a cost of 10½*d*. each. The Association of which we speak stands high in the opinion of the medical men of the North of London, over one hundred doctors having sought the aid of its nurses during the past year.

---

THE Lady Superintendent of a County Hospital writes: "We are sorry to learn that the nursing world will soon lose one of its brightest ornaments in the person of Miss Manson, the clever and energetic Matron of St. Bartholomew's Hospital, who is shortly to be married to Dr. Bedford Fenwick. We must congratulate this gentleman on having chosen such a matrimonial prize. We are given to understand that the attachment is an old one, of nearly eight years' standing."

